# Hematopoietic differentiation: a coordinated dynamical process towards attractor stable states

**DOI:** 10.1186/1752-0509-4-85

**Published:** 2010-06-16

**Authors:** Nadia Felli, Luciano Cianetti, Elvira Pelosi, Alessandra Carè, Chang Gong Liu, George A Calin, Simona Rossi, Cesare Peschle, Giovanna Marziali, Alessandro Giuliani

**Affiliations:** 1 Department of Hematology, Oncology and Molecular Medicine Istituto Superiore di Sanità, 00161 Rome, Italy; 2 Department of Environment and Health, Istituto Superiore di Sanit, 00161 Rome, Italy; 3 Department of Experimental Therapeutics and Cancer Genetics, University of Texas, M.D. Anderson Cancer Center, Houston, TX, USA; 4 IRCCS MultiMedica, 20138 Milan, Italy

## Abstract

**Background:**

The differentiation process, proceeding from stem cells towards the different committed cell types, can be considered as a trajectory towards an attractor of a dynamical process. This view, taking into consideration the transcriptome and miRNome dynamics considered as a whole, instead of looking at few 'master genes' driving the system, offers a novel perspective on this phenomenon. We investigated the 'differentiation trajectories' of the hematopoietic system considering a genome-wide scenario.

**Results:**

We developed serum-free liquid suspension unilineage cultures of cord blood (CB) CD34^+ ^hematopoietic progenitor cells through erythroid (E), megakaryocytic (MK), granulocytic (G) and monocytic (Mo) pathways. These cultures recapitulate physiological hematopoiesis, allowing the analysis of almost pure unilineage precursors starting from initial differentiation of HPCs until terminal maturation. By analyzing the expression profile of protein coding genes and microRNAs in unilineage CB E, MK, G and Mo cultures, at sequential stages of differentiation and maturation, we observed a coordinated, fully interconnected and scalable character of cell population behaviour in both transcriptome and miRNome spaces reminiscent of an attractor-like dynamics. MiRNome and transcriptome space differed for a still not terminally committed behaviour of microRNAs.

**Conclusions:**

Consistent with their roles, the transcriptome system can be considered as the state space of a cell population, while the continuously evolving miRNA space corresponds to the tuning system necessary to reach the attractor. The behaviour of miRNA machinery could be of great relevance not only for the promise of reversing the differentiated state but even for tumor biology.

## Background

The hematopoietic system is a privileged observatory for the study of the highly coordinated process leading to differentiated cells. Hematopoietic proliferation and differentiation is sustained by a pool of multipotent self-renewing hematopoietic stem cells (HSCs), which give rise to a hierarchy of progenitor populations (HPCs) with more restricted lineage potential, ultimately leading to the production of all types of mature blood cells. HSCs/primitive HPCs give rise to a hierarchy of committed HPCs, functionally defined as burst-forming units (BFUs) or colony-forming units (CFUs). The earliest HPCs are multipotent and generate mixed colonies (CFU-GEMM: CFU-granulocytic, erythroid, macrophage, megakaryocyte). Multipotent HPCs differentiate and become gradually committed to specific lineages, i.e., HPCs of the erythroid series (early and late BFU-E and CFU-E) the megakaryocytic lineage (BFU-MK and CFU-MK) and the granulo-monocytic lineage (CFU-GM, CFU-G, CFU-M) [1]. HSCs/HPCs differentiation is controlled by the combined effects of hematopoietic growth factors (HGFs), chromatin modifiers, transcription factors and microRNAs (miRNAs). MiRNAs are a new class of small non coding RNAs (~22 nucleotides), playing a key role in post-transcriptional regulation of gene expression [2,3]. This occurs through degradation or translational repression of target mRNAs, by binding to their 3'-untranslated regions [4,5]. The human genome encodes ~700 miRNAs http://microrna.sanger.ac.uk, located in introns, exons or intergenic regions [6,7]. Several studies have demonstrated that miRNAs have unique developmental-specific expression or signature, since each tissue/cell type produces a specific set of miRNAs [8,9].

Beside the enumeration of the different actors playing in the differentiation act, we have not to forget that the differentiation process can be considered as a trajectory towards an attractor (i.e. a specific cell type), interesting the cell as a whole with a coordinated change in metabolism, gene expression, shape, general responsiveness to environmental stimuli. In this view, the attention is no more focused on a single 'average' cell behavior with a sequential differential gene activation/repression program, but on a mutually interacting ensemble of cells in which the entire transcriptome pattern moves from the initial unstable attractor state (progenitor cells) towards another stable, end attractor, state (mature cells) [10]. By analyzing both protein coding genes (PCGs) and miRNAs expression during hematopoiesis, we gave a proof-of-concept of this attractor-like dynamics and were able to characterize the coordinated and scalable character of cell population behaviour in both mRNAs and miRNAs spaces. These results allow to envisage a sort of biological statistical mechanics along the lines described by Bar-Yam and colleagues [11], in which the traditional "master genes" driven approach may be substituted by a global motion of the system as a whole.

We will also discuss the complementary role of transcriptome and miRNome and describe the miRNome as a sort of continuously adjusting control device of the cell population [12].

## Results

To investigate the cellular and molecular mechanisms underlying haematopoiesis, serum-free culture systems were developed for unilineage differentiation and maturation of cord blood CD34^+^HPCs through the erythroid (E), megakaryocytic (MK), granulocytic (G) or monocytic (Mo) pathways. The CD34^+^HPCs population is an heterogeneous population containing: i) very few HSCs (<0.1%), ii) minority of pluripotent HPCs (1-3%) and iii) majority (>90%) of committed progenitors (i.e. BFU-E, BFU-MK, CFU-GM). In these cultures, HPCs are stimulated by specific unilineage growth factors (HGFs) at a saturating level combined with appropriate dosages of multilineage or early acting HGFs (see material and methods). It has to be considered and is currently accepted that HGFs are permissive and not instructive molecule, thus addition of HGFs mainly induce proliferation, differentiation and maturation of unilineage committed progenitors. Therefore this type of culture allows to analyze 'pure' unilineage precursors at discrete sequential stages of differentiation/maturation process starting from committed progenitors, but do not allow to investigate early stages of hematopoiesis, such as cell fate decision and/or commitment of pluripotent HPCs.

Specifically, unilineage cultures of cord blood CD34^+^HPCs are derived by culturing purified HPCs, separated by positive selection immunomagnetic system for CD34^+^, in serum-free liquid suspension and induced to a) selective E growth by very low dosages of IL-3, GM-CSF, and a saturating erythropoietin (Epo) level, b) unilineage MK growth by saturating dosage of thrombopoietin (Tpo), c) unilineage G growth by low dose of IL-3 and GM-CSF combined with plateau level of G-CSF, and d) unilineage Mo by low dose of IL-6 and saturating dosage of Flt-3 and M-CSF.

**Figure 1:**
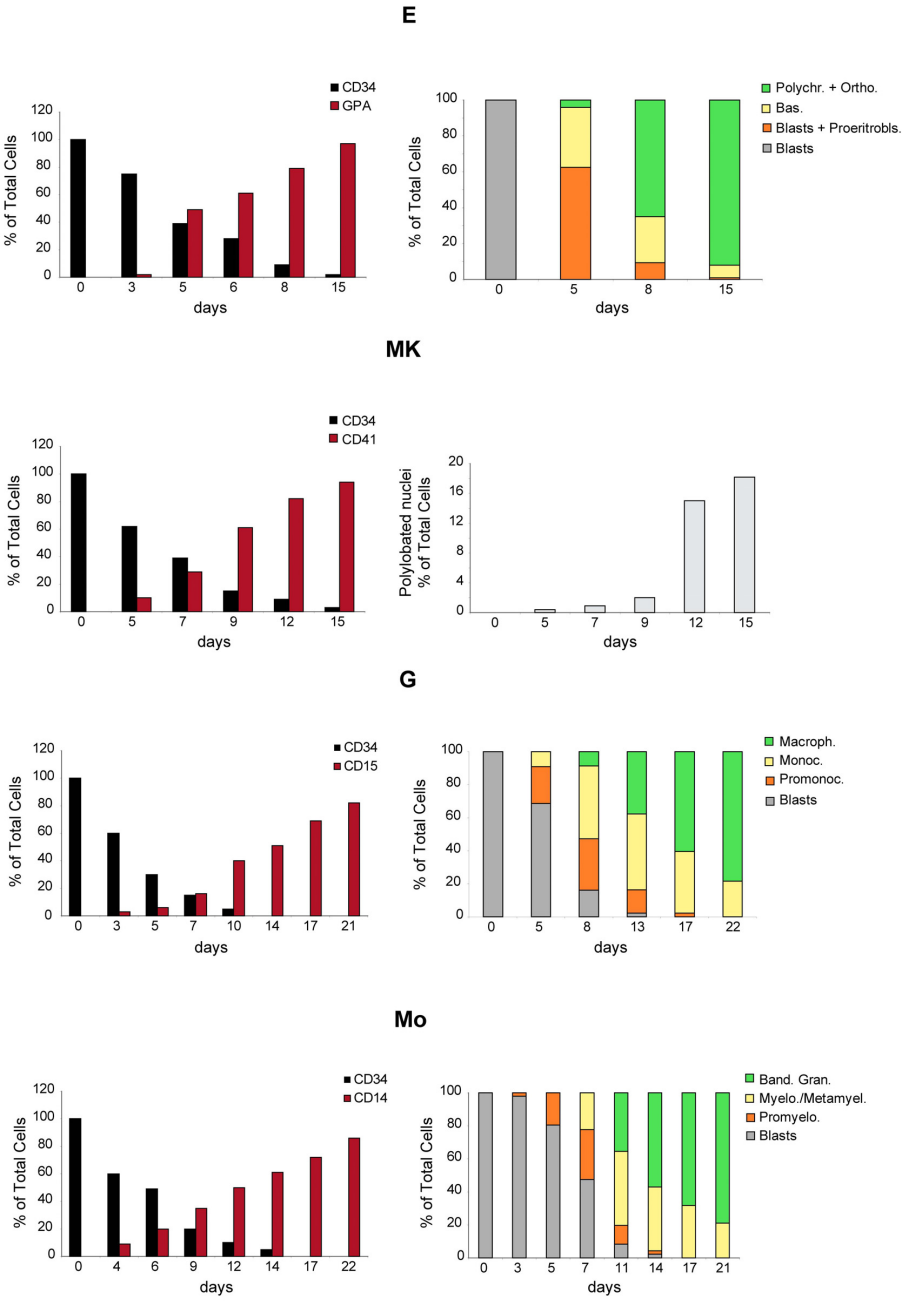
**Growth and differentiation of cord blood (CB) CD34^+ ^hematopoietic progenitor cells (HPCs)**. CB CD34^+^HPCs unilineage cultures allow the analysis of 90-95% pure unilineage precursors as assessed by flow-cytometry and morphology. *(left) *Percentage of GPA, CD41, CD15 and CD14 positive cells in unilineage erythroid, megakaryocytic, granulocytic and monocytic differentiation and maturation. *(right) *Schematic representation of May-Grünwald-Giemsa staining of unilineage erythroid, megakaryocytic, granulocytic and monocytic cultures at various differentiation stages. Percentage of blasts, differentiating and mature cells with respect to total cells is indicated. Representative experiments are presented.

The purity and the differentiation process of each type of these cultures was assessed by flow-cytometry: a gradual decrease of CD34^+ ^marker during the first days of culture, followed by a progressively increasing expression of specific membrane markers (glycophorin A, CD41, CD15 and CD14, respectively) (Figure 1) was observed. Consistently, cell morphological analysis showed a gradual wave of maturation along the diverse differentiative pathways to terminal mature cells (Figure 1).

These culture systems, moving from committed progenitors to mature cells state, allow to analyze the existence, if any, of developmental trajectories during hematopoietic differentiation. To this end, we decided to selectively analyze the expression profiles of PCGs and miRNAs in unilineage culture of cord blood (CB) CD34^+ ^cells (HPCs) through the erythroid, megakaryocytic, granulocytic and monocytic differentiation/maturation. Total RNA was extracted from CD34^+ ^HPCs and at different days of culture, corresponding to discrete sequential stages of differentiation/maturation process, from each type of culture.

Microarray analysis was performed using the GeneChip Human Genome U133 Plus 2.0 oligonucleotide microarrays (Affymetrix) for gene expression profiling, and a custom in-house manufactured miRNA microarray platform (A-MEXP-620 version 3.0) to profile the expression of miRNAs (see additional files 1 and 2: Tables S1 and S2 and materials and methods for accession number).

**Figure 2:**
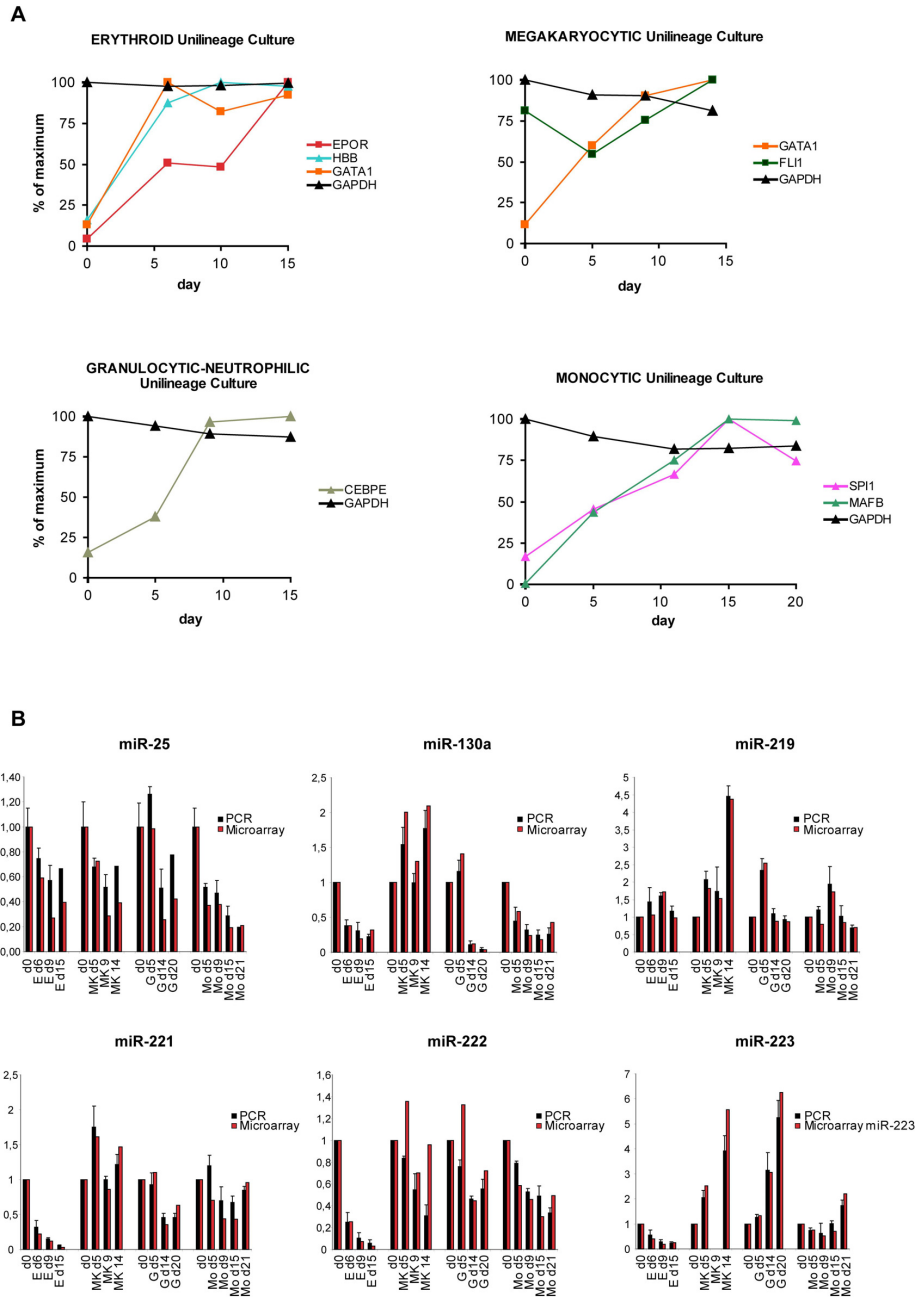
**Expression trend of lineage-specific genes during differentiation of CB CD34^+ ^and miRNA microarray data confirmation**. **(A) **The reliability of gene microarray results was inspected by comparing the variations in signal of well-known lineage-specific genes for each unilineage cultures. The expression of the selected genes was concordant with the expected protein modulation during differentiation/maturation. **(B) **Real-time reverse-transcription polymerase chain reaction of six randomly chosen differentially expressed miRNAs in erythroid, megakaryocytic granulocytic, and monocytic unilineage cultures. qRT-PCR results are compared to miRNA microarray derived values.

The reliability of gene microarray results was inspected by comparing the variations in signal of well-known lineage-specific genes and the corresponding antigens/proteins as reported in current literature [13,14]. In particular, the expression trend was evaluated for the above specific markers, plus other specific proteins such as erythropoietin receptor (EPOR), β-hemoglobin (HBB) and GATA1 for E unilineage [15], Spi1, MAFB and CEBPE for G and Mo cultures [16], GATA1 and FLI1 for MK lineage [17]. Variation in the RNA expression of these markers as reported by microarray was always concordant with the expected protein modulation during differentiation (Figure 2A).

To validate miRNAs expression profile, six randomly chosen differentially expressed miRNAs were analyzed by real-time reverse-transcription polymerase chain reaction (RT-PCR) (Figure 2B). Moreover, several miRNAs showed comparable expression with previously reported trends (i.e. miR-221/222 [18], miR-451, miR-155 during erythropoiesis [19], miR-223 during granulopoiesis [20], and miR-17-5p, miR-20a, miR-106 during monocytopoiesis [21]). Regarding miRNAs expression profile during megakaryopoiesis, we noticed an incomplete overlap between our and previously published results [22,23] possibly due to the different CD34^+^HPCs source (i.e. cord blood versus bone marrow).

In order to understand how PCGs and miRNAs expression patterns correlate with hematopoietic cell differentiation, both genome and miRNAs expression profiles were analyzed as global patterns by using the Gene Expression Dynamics Inspector (GEDI) software [24]. GEDI displays a global picture of the state vector relative to both transcriptome and miRNome monitoring the cell population while it moves towards its differentiation fate.

Genome-wide expression profile of PCGs was representative of a redundant set of 54630 human sequences, with expression signal linear values ranging from 0.293 to 71337.

**Figure 3:**
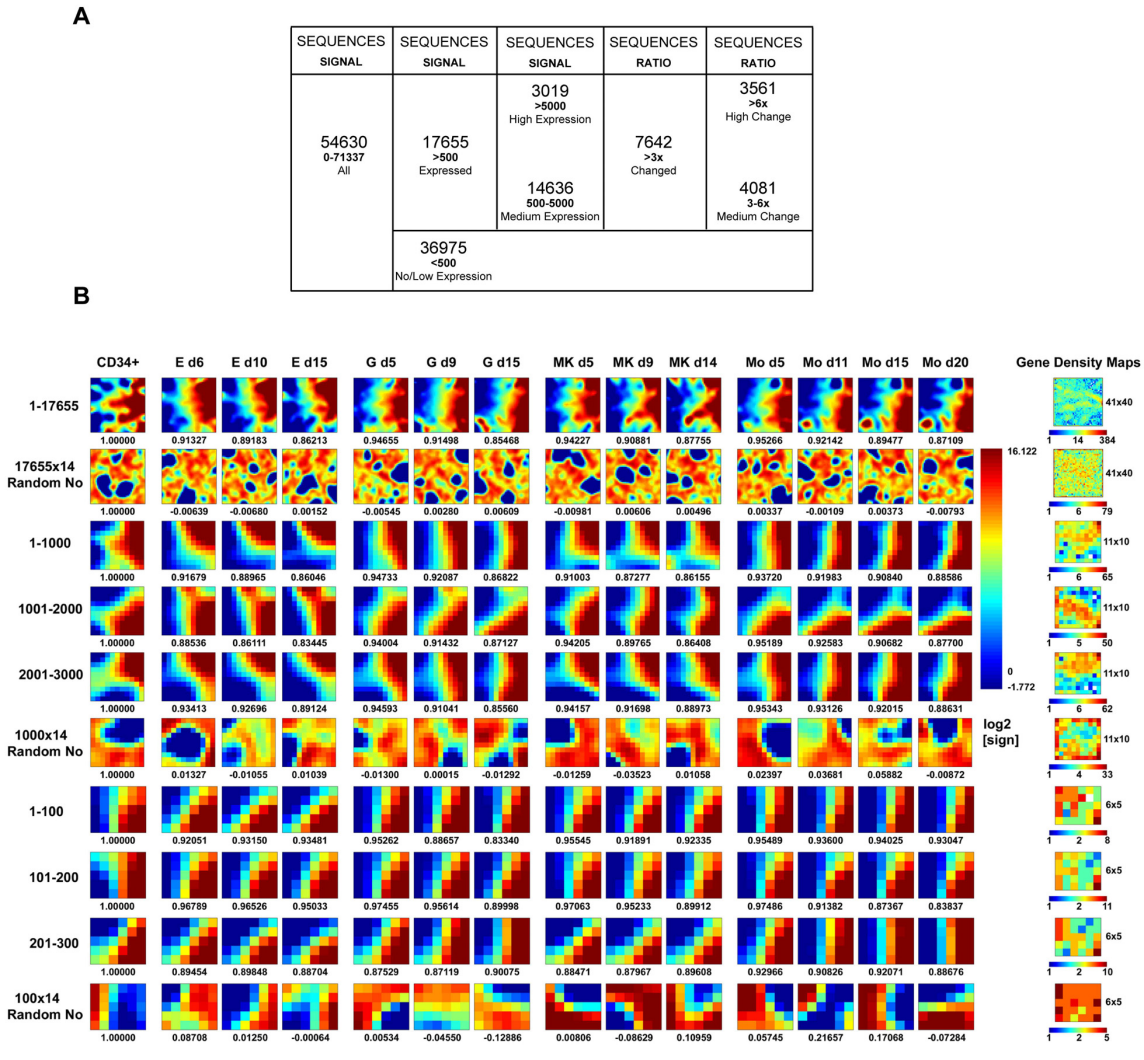
**Expression profile of protein coding genes (PCGs) in unilineage culture of cord blood HPCs**. **(A) **Gene microarrays were representative of a 54630 redundant set of sequences having linear normalized signal values ranging from 0 to 71337. Only sequences having at least one value >500 (an under-expression arbitrary threshold roughly corresponding to 1% of maximum value) were considered significantly expressed (Expressed Sequences) and sub-grouped according to two different criteria: i) expression level, by using a second arbitrary threshold (5000) in signal values that results in Medium Expressed (maximum value in the range 500-5000) and High Expressed (at least one value >5000) genes; ii) fold increase/decrease calculated as the ratio of differentiated cells (t_max_) to CD34^+^HPCs (t_0_) signal, obtaining Changed, Medium Changed and High Changed subsets of sequences showing in at least a lineage a signal fold change >3×, 3-6× or >6× respectively. **(B) **Self-organizing maps of global gene expression for the E, G, MK and Mo differentiation pathways. The array data set of N = 17655 Expressed Sequences, multiple randomly sampled 1000 or 100 sequence subsets and same-sized Random Numbers sets were clustered using the GEDI software into nearly-squared grids composed by miniclusters. On the left data or number set analysed (progressive number) for each line is defined, on the top of each column lineage and culture time are indicated. Below each grid the Pearson correlation with CD34^+ ^(or with the first sample for Random Numbers) is reported. In the grids each pixel corresponds to a minicluster that is located at the same position in all the grids of the same line and is composed by a variable small group of sequences sharing a similar expression pattern, whose number is indicated by the Gene Density Map. On the right grid size (pixel number) is indicated. Colour of each minicluster indicates the mean of log_2_-transformed expression value of corresponding sequences or Random Numbers, as indicated on the column bar on the right.

For GEDI analysis we used a subset of 17655 sequences (Expressed Sequences) having signal values >500 (an under-expression-threshold roughly corresponding to the 1% of maximum expression) in at least a sample (Figure 3A). Resulting representations revealed that after the first step of unilineage differentiation (from CD34^+ ^to day 5-6 of culture) a distinctive lineage-specific gene expression profile was already recognizable. After this time minor changes occurred along the maturation processes to further define the differentiated expression profile (Figure 3B). Earliness of definition of recognizable lineage-specific patterns supports the idea that the mature differentiated cells can be considered as multidimensional attractors in the global expression space [25].

In dynamical terms, the stem/progenitor cells can be considered as an unstable state (unstable attractor) from where the system exits following perturbations (i.e. growth factors, cell-cell interaction), and migrates along the different trajectories in the multidimensional transcript profile space (state space) until reaching the stable ones (stable attractors), corresponding to the mature phenotypes. The transcript profile space is defined by the gene expression pattern; changes of the transcript profile results in changing the position of the cell population in the multidimesional space.

**Figure 4:**
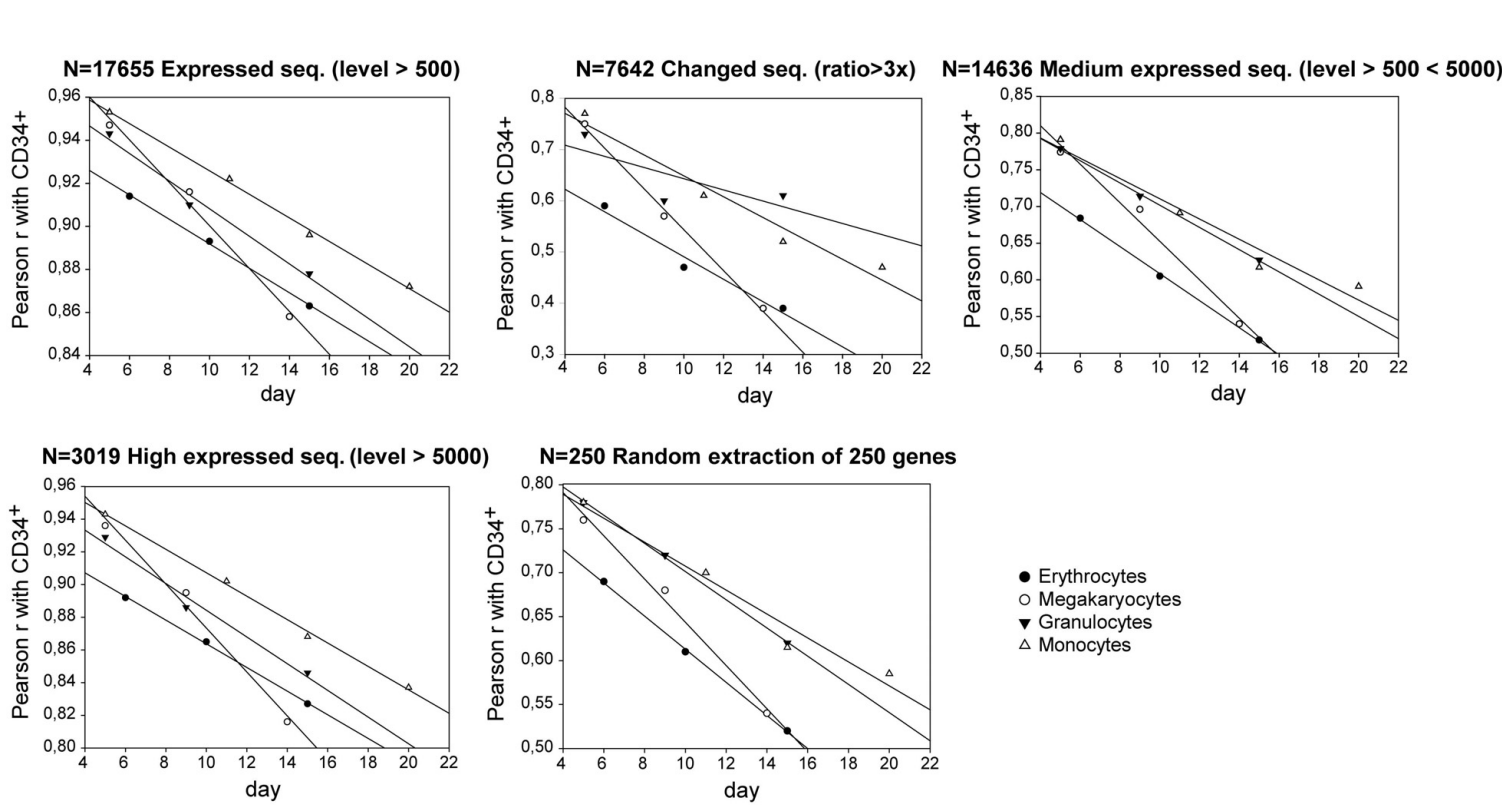
**Correlation between HPCs and hematopoietic cells at different stages of differentiation**. The different panels report the Pearson correlation (r) between CD34^+ ^progenitor cells and the different lineages at subsequent differentiation time points. The progressive advancement of differentiation provokes a linear decrease of correlation in time. The substantial homogeneity of the graphs with different gene subsets points to the scalable character of the trajectories towards the final attractors.

We then calculated the global similarity between samples on the entire expressed genome (17655 Expressed Sequences) in terms of Pearson correlation (r). As expected, given we are dealing with samples of the same general cell-type, genome-wide correlations are extremely high (Pearson r around 0.90), indicating a strong common order parameter influencing the expression level of the entire genome (Figure 4). Interestingly, we observed a monotonically decreasing correlation coefficient between CD34^+^HPCs and different lineages at increasing time, consistent with the increasing distance from the initial unstable stem/progenitor cell attractor. To further analyze the attractor like properties of the differentiation dynamics we calculated the first derivative of the trajectory change in terms of corr(t1) - corr(t0)/(t1 - t0), being t1 and t0 two different times of observations and corr (t) the relative correlation with initial state. As evident in Figure 3, the decrease in correlation from unit value is concentrated on the first time step, being the subsequent decrease in time much less pronounced consistently with a progressive decreasing of the derivative (data not shown). This flattening of the differentiation rate appears to be particularly pronounced in the case of erythroid differentiation, in which the major part of total displacement from initial state is accomplished in the first time step.

In order to demonstrate the scalability of gene expression dynamics (i.e. the possibility to get the same general picture of differentiation dynamics adopting different size views of the genome) different gene sets were selected. The 17655 Expressed Sequences were divided in 14636 Medium Expressed (maximum signal in the range 500-5000), 3019 High Expressed (signal >5000 in at least one sample) and 7642 Changed (signal variation >3× in respect to CD34^+ ^in at least a sample) sequences subsets (Figure 3A).

The Expressed Sequences set gave exactly the same results in terms of monotonic linear decrease of correlation with CD34^+^HPCs pattern of all the other choices (Medium Expressed, High Expressed and Changed Sequences). The same progressive displacement from unstable CD34^+^HPCs attractor is highlighted with the Changed Sequences and with a random 250 genes extraction (Figure 4).

The same invariant character of displacement from progenitor cell line was observed in further subdivided sets (i.e. signal values >500 with 6-fold change between CD34^+^HPCs and mature cells) (data not shown).

Another proof-of-concept of the scalable character of differentiation process came from GEDI analysis performed on multiple subsets of 1000 or 100 sequences randomly picked up from the Expressed Sequence set (Figure 3B). The smallest subsets (100 genes) too showed stable, lineage-specific expression patterns, even if with a less monotonically decreasing correlation trend from CD34 with respect to bigger sets.

These results demonstrate the scalable character of the progressive displacement of the different lineages from their common stem/progenitor cells. Therefore, we can consider these analyzed cell populations as strongly connected dynamical systems in which the expression of different genes is highly correlated [26,11].

**Figure 5:**
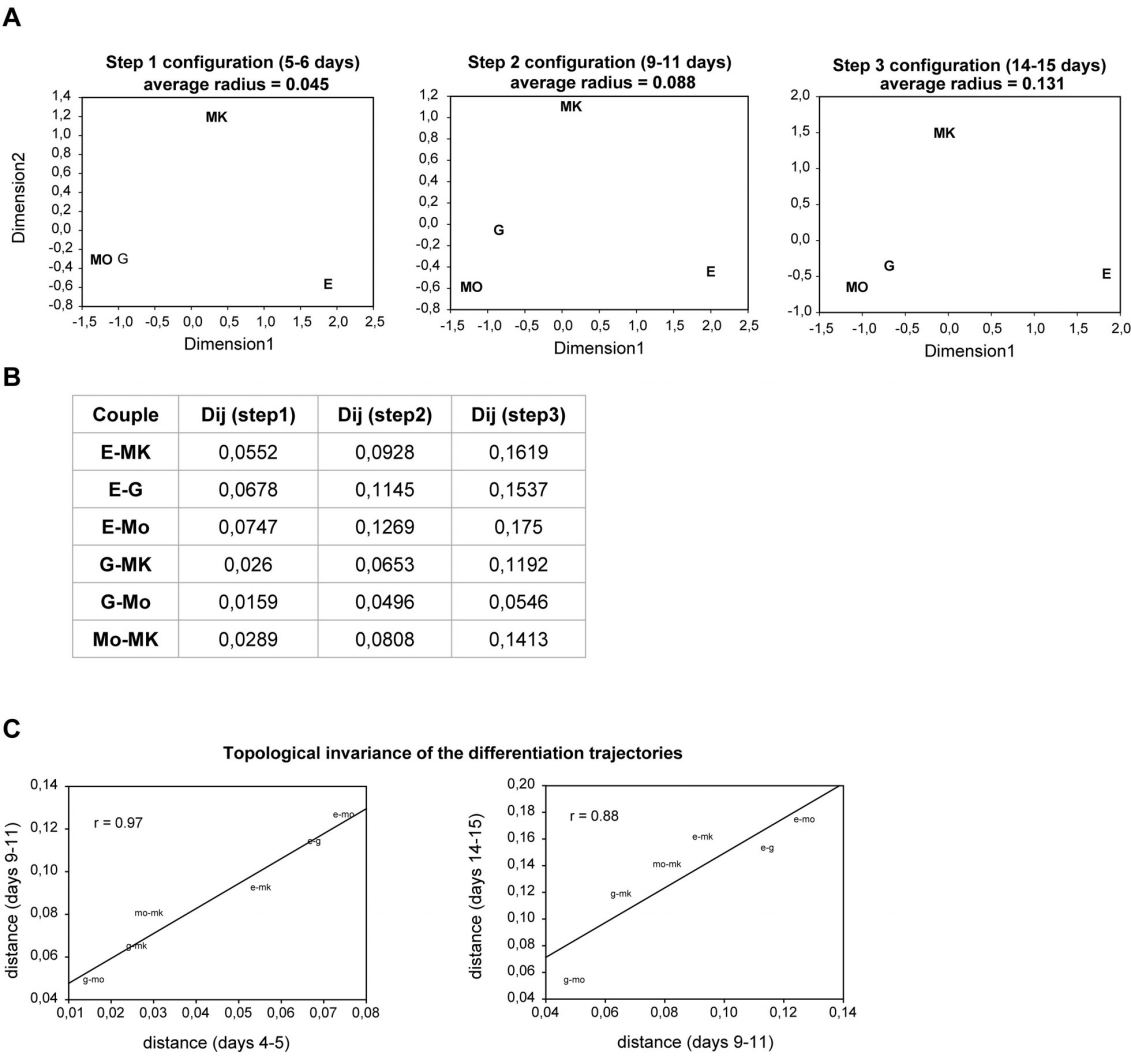
**Progressive displacement of different cell lineages from unstable state (stem/progenitor cells) to attractors (differentiated cells)**. **(A) **Multidimensional Scaling plots of the among lineages distances as computed on the entire transcriptome at different steps of differentiation. The average radius of the entire set increases in time consistently with the progressive differentiation of lineages, the relative distances between samples remain linearly invariant (i.e proportional), pointing to an early commitment of the lineages at five days and to a common differentiation rate. **(B) **Distances between lineages determined by computing their genome-wide mutual correlations at different time points. **(C) **Correlation between pairwise lineage distances at different time windows. The scoring of a linear correlation between pairwise samples distance is the proof of a progressive motion along a smooth differentiation landscape.

Having stated that all the diverse differentiation trajectories monotonously move from their progenitors, we asked whether the mutual relations between different cell populations during the trajectory towards their fates are conserved. This corresponds to check for the maintaining of a stable differentiation rate across cell lines, so that their mutual distances (in terms of 1-r metrics with r the Pearson correlation coefficient), even if increasing during differentiation process, maintain their relative proportions. In order to answer this question, we used a multidimensional scaling (MDS) technique (Kruskal-Wish scaling), that allows for the construction of a bi-dimensional plot where the relative distances of the objects mirror the effective distances of the same objects computed over the multidimensional space, constituted by their expression levels over the entire genome (Figure 5A) [27]. We computed such distances (Figure 5B) in three distinct temporal windows denominated step 1, step 2 and step 3 corresponding to 5-6, 9-11 and 14-15 differentiative days, respectively (Figure 5A). As shown, the relative position of the lineages remains strongly invariant during the entire time span analyzed so pointing to: i) the existence of distinct attractors towards which the gene expression dynamics of the cells are directed starting from day 5; ii) the progressive enlargement of the phase space spanned by the different cell kinds with the progression of the differentiation process, as marked by the increasing average radius correspondent to the increase of the between lineages distances as for expression patterns along an already decided and fixed direction. Thus, the distance between cell kinds increases as a consequence of the progression of differentiation process, but the relative positions of the different cell kinds are maintained. This indicates that the system continues along the same kind of trajectory, maintaining the overall shape that can be considered as the phase space of the hematopoietic differentiation process. The topological invariance of MDS space, even if not a conclusive proof, strongly points to the existence of a specific attractor for each cell kind in the transcriptome space. The distance space has a very important characteristic: the ability to detect the change of 'shape' of the studied object without the need of making any unjustified assumption. A linear trajectory maintaining its end-point (attractor) implies the increasing of size of the object (the data set made by the 4 lineages) without any modification in shape and thus maintaining the relation between different cell kinds positions linearly invariant. The distances between cell kinds monotonically increase in time but maintain invariant their relative proportions and thus the relative positions. As a consequence the distances between the trajectories are correlated in time (see figure 5, panels B and C).

Having stated the genome-wide expression is a suitable space for describing the differentiation trajectories of blood cell lineages, we turned our attention to the miRNA space.

**Figure 6:**
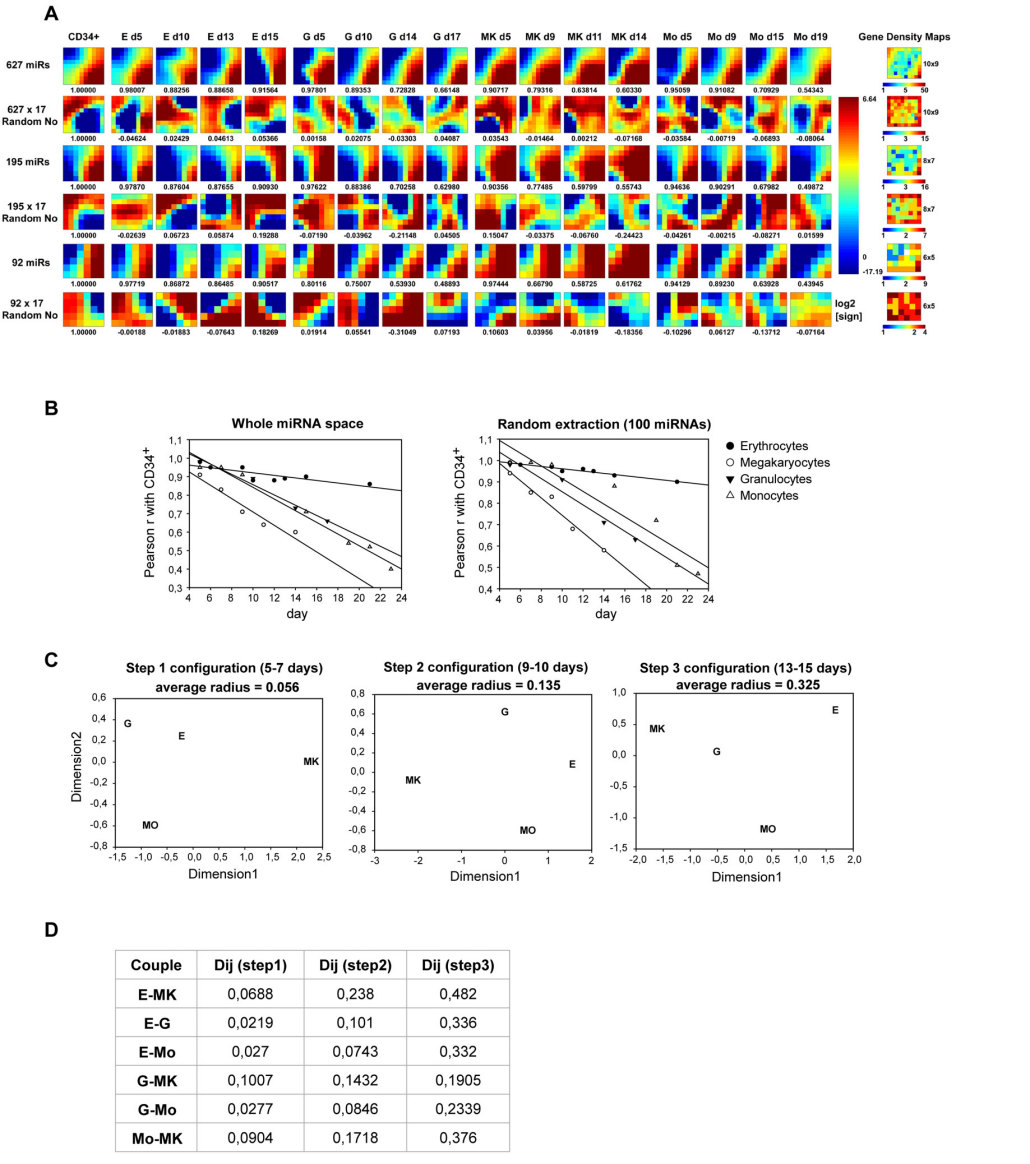
**miRNAs space in unilineage culture of cord blood HPCs**. **(A) **Self-organizing maps of entire microRNA space for the E, G, MK and Mo differentiation pathways. The miRNAs data sets (N = 627 all miRNAs analysed, N = 195 and N = 92 miRs having in at least a sample signal >1% or >4% respectively of maximum value) and same-sized Random Numbers sets were clustered using the GEDI software into nearly-squared grids composed by miniclusters. On the left, data set analysed for each line is defined, on the top of each column lineage and culture time are indicated, below each grid the Pearson correlation with CD34^+ ^(or with first sample for Random Numbers) is reported. In the grids each pixel corresponds to a minicluster that is located at the same position in all the grids of the same line and is composed by a variable small group of miRNAs sharing a similar expression pattern, whose number is indicated by the Gene Density Map. On the right grid size (pixel number) is indicated. Colour of each minicluster indicates the mean of log_2_-transformed expression value of corresponding miRNAs or Random Numbers, as indicated on the column bar on the right. **(B) **Progressive displacement in time measured in terms of Pearson correlation (r) with CD34^+ ^profile, as computed across the entire microRNA space (top) and on a random extraction of microRNAs. **(C) **Multidimensional Scaling plots of the among lineages distances, as computed on the microRNA space at three different steps of differentiation. The average radius increases in time consistently with the progressive differentiation of lineages, while the relative distances between samples do not remain linearly invariant. **(D) **Distances between lineages determined by computing their miRNA mutual correlations at different time points.

The GEDI analysis of 627 miRNAs exhibits clear distinct expression patterns associated with different hematopoietic lineages, revealing distinctive signatures for each hematopoietic differentiative pathway (Figure 6A). Moreover defined, correlated and lineage-specific expression profiles were also obtained using subsets including 195 and 92 miRNA having in at least a sample signal >1 or >4% respectively of maximum signal.

In analogy with the transcriptome space, we observed a progressive linear displacement of miRNA patterns from the HPCs (Figure 6B), as shown by the trend of Pearson correlation coefficient of the four different lineages with CD34^+^HPCs population. These results were confirmed by analyzing a replicate microarray (additional file 3, Figure S1).

The scalability feature, observed in the transcriptome, is present in the miRNAs space as well: the analysis of a randomly extracted sample of 100 miRNA sequences (Figure 6B right) gave us exactly the same results of the full-rank space (627 miRNAs, Figure 6B left). The analysis performed with less than 50 randomly picked-up elements do not anymore constitute a reliable sampling giving rise to widely varying results from sample to sample. The scalability of microRNA regulation tells us that even this system 'acts as a whole' being a connected system with several mutual correlations between its elements.

The MDS reveals a profound difference with the transcriptome based picture (Figure 6C), showing that the topology of the miRNA space is much less invariant than the correspondent transcriptome space. The lack of invariance is mirrored by the absence of any linear relation between the mutual distances among lineages at different steps (r = -0.013 between step 1 and step 3 distances, Figure 6D) in the miRNA space. The difference between micro RNA and transcriptome spaces in terms of topological invariance of the mutual relations between cell kinds profiles is not due to the smaller number of variables considered for miRNome. As a matter of fact, the MDS based portraits computed over six different 627 genes random extractions gave completely coincident results with the ones obtained on the whole set (additional file 4, Figure S2). The mutual distances between the cell lineages computed at different steps along the development trajectory (steps 1, 2 and 3 as depicted in the additional file 4, Figure S2) where remarkably invariant between the whole set and random extractions with an average correlation between distance matrices r = 0.93. This gave rise to completely coincident MDS portraits. This result indicates that, while, in terms of transcriptome state, a cell population can be considered already committed to its fate at five days of differentiation and consequently following a differentiation trajectory with a fixed orientation as described by MDS, the miRNA pattern shows a still not finally committed shape until the end of the studied period.

We can speculate on this lack of 'orientation invariance' of miRNA space, stressing the fact that while genome wide expression profile marks the 'average state' of the cell population at a given instant of time, the miRNA pattern can be equated to the 'fine controls' acting on the system to drive the trajectory towards its differentiation fate and actively maintaining the population on the attractor [12]. In other words while the transcriptome is the 'location in space' of the system, the miRNA machinery, for its regulative role, can be considered as its 'instantaneous velocity vector'.

## Discussion and Conclusions

The hematopoietic differentiation process permits to follow the dynamic of a system when moving from a common unstable progenitor state to different stable attractors correspondent to the distinct cell types, each characterized by a clearly distinguished profile in the multidimensional transcript profile space.

Our study showed that both transcriptome and miRNome gene expression profiles map cells into a multidimensional space, where the positions of differentiating cells progress along trajectories starting from undifferentiated CD34^+ ^cells towards specific differentiation fates, in line to the existence of the attractor-like dynamics during hematopoiesis.

The transcriptome and miRNome spaces derive from the expression patterns of protein coding genes and miRNAs respectively, and coordinated changes in their expression patterns can be interpreted as trajectories of cell populations in a multidimensional space. The analysis of gene and miRNAs expression profiles gave a proof-of-concept of the scalable character of biological regulation circuits. While the increasing distance (i.e. the progressive decay of Pearson r) representing the increasing differences between the various differentiating cell types and the progenitor cells appears as an almost trivial result, it is important to highlight that the same behaviour is observed with widely different choices of gene subsets, including those genes that are routinely considered as 'noisy and unreliable information'. This implies the strong connectivity of the gene regulation network that can be considered as a unitary system so casting doubts about classical concepts like the existence of 'housekeeping genes' whose expression is largely invariant and decoupled from specific cell activity. As a matter of fact scalability (i.e. the possibility to observe the same general behaviour at different scales, here corresponding to different choices of genes) is a very important consequence of the statistical-mechanics like behaviour of cell populations that until now was only marginally taken into account [26,28]. Under this paradigm the principal actor of the biologically relevant process is no more the single cell, but a cell ensemble which different sources of noise shape as a distribution of elements (cells) slightly differing between them in analogy with Maxwell-Boltzmann distribution of kinetic energy over an ensemble of particles. The energy landscapes typical of physical systems are mirrored, in the case of cell populations, by the so called epigenetic landscapes [29].

The proportionality of the mutual distances between different cell populations during the trajectory toward their fates was observed for embryonic stem cells by Aiba and colleagues [29], by using the epigenetic landscape model proposed by Conrad Waddington [30]. During development, the epigenetic landscape is intended as a physical landscape made of hills and valleys in which a development trajectory is like a marble rolling according to the landscape shapes it encounters. In this model, the unstable stem cell population occupies the top of an hill, from which the system can escape following the different faces of the hill. In this case, two trajectories going along two different directions and remaining with the same bearing (driven by the same topology of the hill) while being progressively set apart between them, will maintain the same relative mutual orientation during all the process. We observed a similar dynamics by analysing unilineage HPCs differentiation using a multidimensional scaling technique: the distances between different cell lines, while increasing in time, maintain their mutual correlation/orientation in the transcriptome space. This correlation is no more present in the miRNA space. The dynamics of differentiation/maturation in the gene expression space has many features consistent with an attractor-like behaviour. Our experimental conditions do not allow for the ultimate proof of the existence of a proper attractor, i.e. the robustness of the differentiated cells to external perturbations, but the obtained results globally support the idea of attractor.

Both transcriptome and miRNome dynamics showed a scalable character and progressive displacement from stem/progenitor cells of the four different lineages during the differentiation process, but while the transcriptome trajectories conserved the relative relations between cell kinds distances this was not the case in the miRNA space.

This behaviour is consistent with the view of the transcriptome as a state space of a cell and miRNA space as a sort of "continuously adjusting" tuning system, affecting cell lineage evolution to reach the attractor.

The reconciliation of transcriptome and miRNome findings is not an easy task. We tried and look for a simple transcriptome-miRNome relation by computing the enrichment in hematopoietic related genes in the targets of miRNAs that vary significantly during the differentiation process. The enrichment level did not differ from a purely random choice of miRNAs (data not shown), thus not allowing for a single miRNome-transcriptome relation to be drawn. This lack of a simple correspondence between regulated genes and miRNA profile is consistent with the holistic character of attractor-like dynamics described. Moreover, these results are in line with the results of Liu and Kohane [31] and with the observation of Seitz H. [32] which propose that many computationally identified miRNA targets may actually be competitive inhibitors of miRNA function, preventing miRNAs from binding their authentic targets by sequestering them. Thus we must limit ourselves to the registration of a different dynamics of global miRNome with respect to transcriptome without going further into unjustified speculations.

Furthermore, our data do not allow to analyze whether or not cell population arrives at a more or less stable miRNA profile, anyway we believe that a relevant and continuously on-going tuning of the system by the miRNAs is needed to actively maintain the differentiated state. Moreover, considering that miRNAs represent a fundamental epigenetic regulatory mechanism, their behaviours suggest the possibility of their involvement in reprogramming or reversing differentiated cells. Reprogramming is the activation of a pre-existing gene expression program caused by stimulating a transition into the attractor that encodes such a program. MiRNAs modulation may be a reasonable way connecting the attractors according to Huang model [12].

The discovery of these dynamics in miRNA space should be of great relevance not only for the promise of reversing the differentiated state, but even for tumor biology. As a matter of fact while the differentiation process is the approaching to a stable attractor from an unstable one, the process of tumor formation can be considered as the escaping from the stable attractor correspondent to the normal tissue shifting to a less differentiated, embryonic-like mode correspondent to the tumor state. This consideration opens another important research avenue in cell system dynamics.

Our findings may have implications in the interpretative analysis of gene expression signature and possibly in the choice of future therapeutic options for cancer diseases. The consideration of gene expression as a whole was already applied for diagnosis purpose [33], but never on miRNA expression. Another important implication of this way to consider hematopoietic process is that the locations of cells in the multidimensional transcriptome and miRNome space can be used to identify cells of unknown origin, to determine the differentiation status of cells and to assess their multi-differentiative potency.

## Methods

### Cord blood human progenitor cells purification and cultures

Human cord blood was obtained after informed written consent and processed under approval of the Istituto Superiore di Sanità ethics committee.

Isolation of CD34^+ ^cells from cord blood (CB), unilineage culture and morphological analysis were performed as described [34]. Briefly, CD34^+ ^cells were purified from CB by positive selection using the midi-MACS immunomagnetic separation system according to the manufacturer's instructions (Miltenyi Biotec, Bergisch Gladabach, Germany). The purity of CD34^+ ^cells was assessed by flow cytometry using a monoclonal PE-conjugated anti-CD34 antibody and was routinely over 95% (range comprised between 92 and 98%). CD34^+ ^progenitors were cultured in serum-free medium in the presence of various recombinant human cytokine combinations. Serum-free medium was prepared as it follows: freshly prepared Iscove's modified Dulbecco's medium was supplemented with bovine serum albumin (10 mg/ml), pure human transferrin (700 mg/ml), human low-density lipoprotein (40 mg/ml), insulin (10 mg/ml), sodium pyruvate (10^-4 ^mol/l), L-glutamine (2 × 10^-3 ^mol/l), rare inorganic elements supplemented with iron sulfate (4 × 10^-8 ^mol/l) and nucleosides (10 μg/ml each).

For erythroid unilineage culture, serum-free medium was supplemented with 0.01 U/ml IL-3, 0.001 ng/ml GM-CSF (PeproTech Inc. Rocky Hill, NJ, USA) and 3 U/ml erythropoietin (Amgen Thousand Oaks, CA, USA). In these culture conditions, progeny of cells 97 +/- 2% glycophorin-A cells were generated. For megakaryocytic unilineage culture, medium was supplemented with 100 ng/ml thrombopoietin (PeproTech). In these culture conditions, progeny of cells 94 +/- 3% CD41^+ ^were generated. For granulocytic unilineage culture, medium was supplemented with 1 U/ml IL3, 0.1 ng/ml GM-CSF combined with plateau level of G-CSF (500 U/ml) (PeproTech). In these culture conditions, progeny of cells 80-85% CD15^+ ^were generated. For monocytic unilineage culture, serum-free medium was supplemented with 1 ng/ml IL6, 100 ng/ml Flt3-ligand, combined with plateau level M-CSF (50 ng/ml) (PeproTech). In these cultures, progeny of cells 100 +/- 2% CD14^+ ^were generated. For morphological analysis, cells were smeared on glass slides by cytospin centrifugation, stained with May-Grünwald-Giemsa and analyzed at 400× magnification under a microscope (Eclipse 1000, Nikon, Tokio, Japan) equipped with a digital camera.

### Flow Cytometry analysis

Cells were washed and incubated 30 minutes on ice in the dark with 3-5 μg/ml PE conjugated monoclonal antibody to Glycophorin A (GPA), CD34, CD41, CD15 and CD14 or isotype control (Pharmingen, San Diego, CA, USA). Cells were then washed and analysed on a FACSCanto (Becton Dickinson, Franklin Lakes, NJ, USA). Acquisition and analysis were performed using Diva software (Becton Dickinson).

### Microarrays and bioinformatic analysis

To analyze microRNAs expression in human hematopoietic cells, total RNA was obtained using TRIzol Reagent (Invitrogen Life Technologies, Carlsbad, CA, USA). Microarray analysis was performed as described [35,36]. Briefly, labeled targets from 5 μg of total RNA were used for hybridization on microarray chip containing 3766 probes in triplicate, including 627 human precursors miRNA genes. The probes (40-mer oligonucleotides) are spotted by contacting technologies and covalently attached to a polymeric matrix. The microarray were hybridized in 6 × SSPE/30% formamide at 25°C for 18 h, washed in 0.75 × TNT (Tris-HCl/sodium chloride/Tween) at 37°C for 40 min, and processed by using direct detection of the biotin-containing transcripts by Streptavidin - Alexa647 conjugate.

Processed slides were scanned by using Axon 4000 B Scanner (Molecular Device, Orleans Drive Sunnyvale, CA, USA). The expression level were analyzed by GenePix Pro 6.0.0.74 software.

Raw data were normalized and analyzed using the GeneSpring GX 7.3.1 software (Agilent, Foster City, CA, USA). Expression data were median centered. Statistical comparisons were performed by using the ANOVA (Analysis of Variance) tool, using the Benjamini and Hochberg correction for false positives reduction. Cluster analysis was performed using the Pearson correlation as measure of similarity. Microarray data are conform to the MIAME guidelines and have been deposited in ArrayExpress http://www.ebi.ac.uk/arrayexpress under the accession number E-MEXP-2148.

To analyze mRNAs expression in human hematopoietic cells, total RNA was purified from 1-12 × 10^6 ^cells using RNeasy Mini columns (Qiagen GmbH, Hilden, Germany). RNA was quantified spectroscopically (A_260 _nm) and quality was assessed by evaluating the A_260_/A_280 _ratio and analyzing electrophoretic migration of ribosomal RNAs in 1% agarose gel of a 65°C denaturated 1 μg aliquot. When the presence of genomic DNA was revealed, RNA samples were treated with DNaseI.

Gene expression profiling of 14 different samples, corresponding to 3-4 time points for each of the 4 differentiative pathways, was determined using the GeneChip Human Genome U133 Plus 2.0 oligonucleotide microarrays (Affymetrix, Santa Clara, CA, USA) containing a redundant probe set of 54630 sequences. Sample labeling of a 5 μg RNA aliquot, hybridization, washing and scanning were performed according to standard manufacturer's procedures (Affymetrix). Raw data were normalized by first calculating the mean value of the signal of the housekeeping (HK) β-actin (ACTB), α-tubulin (TUBA1B), GAPDH and ubiquitin C (UBC) genes in each sample and then dividing signal values of each time point (t_i_) for the result of ratio HKmean(t_i_)/HKmean(t_0_). Microarray data are conform to the MIAME guidelines and were made publicly available by submission to ArrayExpress under the accession number E-MEXP-2146.

Genome-wide and miRNAs expression patterns was analyzed by using the Gene Expression Dynamics Inspector (GEDI, version 2.1) program [24,25]. Briefly, this algorithm transforms high dimensional gene expression data into distinct two-dimensional color graphic representations deriving from the averaging over a suitable nearly-squared pixel grids, in which each pixel represents the mean value of a given group of genes (around 5-10 genes/pixel on average) having similar expression pattern among different samples. For GEDI analysis were used the log_2_-transformed linear normalized absolute values, with 20 (1^st ^phase) and 80 (2^nd ^phase) training iterations and a variable grid size (41 × 40, 11 × 10, 10 × 9, 8 × 7 or 6 × 5 according to sequence number) as settings. Sampling of PCGs for scalability analysis was performed on the 17655 Expressed Sequences set by ordering lines corresponding to different sequence ID on the basis of a new-randomly generated numerical column, and subsequently picking up multiple 100 or 1000-sized sequence data sets. For each analysis a negative control was included by applying the same procedure to a same-sized set of numbers randomly generated among a comparable range (1-50000 for PCGs and 1-100 for miRNAs). The global invariance of the aspect of the two dimensional color pattern relative to a given lineage is the image in light of the reaching of an attractor stable state encompassing the entire transcriptome, while the emergent character of the gene expression modification is implicit in the progressive averaging (analogous to the renormalization group of statistical mechanics) procedure. The scalable properties of the system were assessed by different choices of subsets of genes into which perform the analysis.

The analysis of both PCG and miRNA spaces in terms of system dynamics was performed using the Pearson correlation coefficient or product moment correlation. We applied this index in order to have a measure of the level of concordance of any two expression vectors correspondent to two different biological samples, x and y with n dimension (n = genes) and mean values of expressions  and . Their mutual Pearson correlation, *r *= (***x***, ***y***) is then defined as:(1)

where , corresponding to the differences from the mean expression of each gene in the X and Y sample respectively and θ is the angle between two expression vectors. Geometrically, Eq. 1 shows that the correlation coefficient can be viewed as the cosine of the angle on n-dimensional space between the two vectors of data which have been shifted by the average to have mean zero. Angle q is a measure of the differences between the two vectors and consequently of the difference in expression pattern of the two sample, when θ = 0 (and consequently r = 1) the two expression patterns are completely coincident, and the two vectors are parallel. In the case of r = 0 (and consequently θ = 90 degrees) the two expression vectors are orthogonal, i.e. the expression patterns of the two samples are each other independent.

The measure Dij = 1-Rij with R = Pearson correlation coefficient between i and j samples can be considered as a between sample distance going from 0 (R = 1) for the total resemblance of the two samples to 1 as maximal possible distance (given the practical impossibility, in this particular case, of the scoring of a relevant negative correlation).

The Kruskal-Wish Multidimensional Scaling (MDS) technique was adopted to accommodate the studied samples into a bi-dimensional plane, maintaining as invariant as possible the original between samples distance. This technique, like any MDS procedure, projects the samples into an abstract space maintaining the initial between samples distances that in our case derive from the original n-dimensional (with n = number of genes) expression space through the agency of Pearson correlation.

## Authors' contributions

NF carried out the molecular studies and drafted the manuscript, LC carried out the molecular studies, performed the GEDI analysis and drafted the manuscript, EP purified and prepared the unilineage hematopoietic cultures, AC participated in the design of the study, CGL performed microRNA arrays, GAC performed microRNA arrays and participated in the design of the study, SR performed the statistical analysis on the correlation between microRNAs and gene expression data, CP participated in its design and coordination, GM conceived the study, participated in its design and wrote the paper, AG performed the statistical analysis, participated in its design and wrote the paper. All the authors read and approved the final manuscript.

## Supplementary Material

Additional file 1 Table S1: Expression of selected genes during hematopoietic differentiation. Click here for file

Additional file 2 Table S2: Expression of 627 human microRNA during hematopoietic differentiation. Click here for file

Additional file 3 Figure S1: Pearson correlation (r) with CD34^+ ^profile of the four different lineages. Click here for file

Additional file 4 Figure S2: Correlation between whole set distance space and a representative random gene extraction (627 genes). Click here for file
