# Supplementary material for: Hematopoietic differentiation: a coordinated dynamical process towards attractor stable states
Source: BMC Syst Biol. 2010 Jun 16;4:85. doi: 10.1186/1752-0509-4-85 (PMC2904736; doi:10.1186/1752-0509-4-85)

**Supplementary figure 1**

The correlation with CD34^+^HPCs miRNA profile for the different times and cell lineages relative to the replicate experiment are reported below:

| **days** | **E** | **MK** | **G** | **MO** |
| --- | --- | --- | --- | --- |
| **0** | 1 | 1 | 1 | 1 |
| **5** | 0,78 |  | 0,8 |  |
| **8** | 0,67 |  | 0,72 |  |
| **8** | 0,69 |  |  |  |
| **15** | 0,71 |  |  |  |
| **12** |  | 0,74 |  | 0,76 |
| **16** |  | 0,76 |  | 0,78 |
| **18** |  |  | 0,71 |  |
| **25** |  |  |  | 0,79 |

These data correspond to this graph:


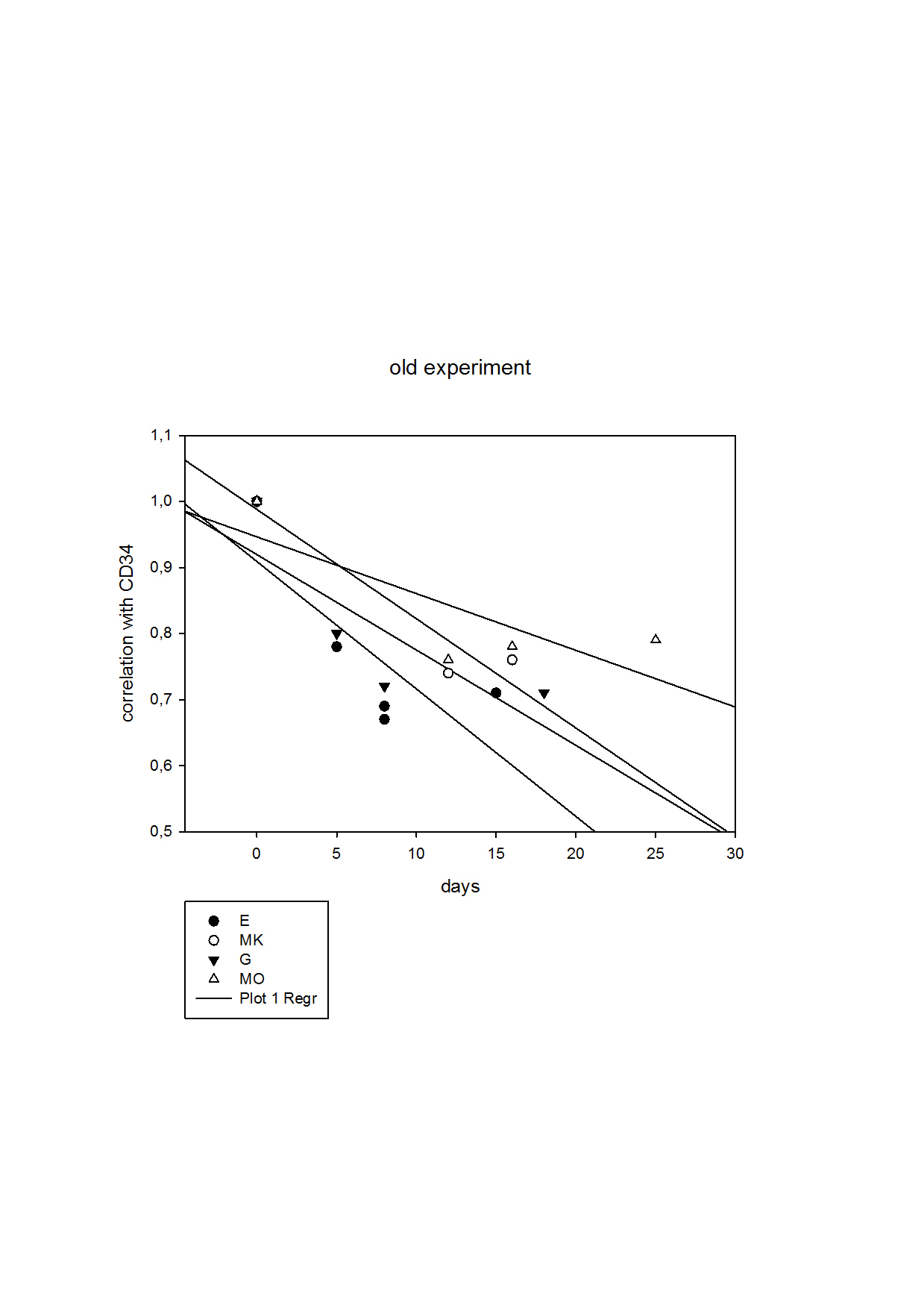

Supplement: Additional file 3 — Figure S1: Pearson correlation (r) with CD34+ profile of the four different lineages. [file 1752-0509-4-85-S3.DOC]
