# Supplementary material for: Hematopoietic differentiation: a coordinated dynamical process towards attractor stable states
Source: BMC Syst Biol. 2010 Jun 16;4:85. doi: 10.1186/1752-0509-4-85 (PMC2904736; doi:10.1186/1752-0509-4-85)
Supplement: Additional file 4 — Figure S2: Correlation between whole set distance space and a representative random gene extraction (627 genes). [file 1752-0509-4-85-S4.DOC]

**Supplementary figure 2**
